# The End-Results of Forty-One Operations for Internal Derangements of the Knee-Joint

**Published:** 1912-03

**Authors:** A. Rendle Short

**Affiliations:** Surgical Registrar, Bristol Royal Infirmary; Senior Demonstrator of Physiology, University of Bristol


### THE END-RESULTS OF FORTY-ONE OPERATIONS FOR INTERNAL DERANGEMENTS OF THE KNEE-JOINT.

BY

A. Rendle Short, M.D., B.S., B.Sc. Lond., F.R.C.S. Eng.,

*Surgical Registrar, Bristol Royal Infirmary;*

*Senior Demonstrator of Physiology, University of Bristol.*

During the ten and a half years from January, 1901, to July, 1911, there have been treated by operation at the Bristol Royal Infirmary fifty-six cases of internal derangement of the knee-joint out of a total of 22,782 surgical in-patients. Of these it has been possible to obtain an after-history in forty-one instances.

The total number of cases may be divided into six classes, according to the record of the condition found at operation.

Class I. *A normal or slightly movable cartilage found, not torn or detached.*—Thirteen cases, with after-history of eight.

Class II. *Cartilage torn, displaced, or very loose.*—Twenty cases, with after-history of seventeen.

Class III. *A loose body found.*—Eleven cases, with after-history of seven.

Class IV. *Condition inadequately described in the notes* (probably most of these should be placed in Class I).—Six cases, with after-history of five.

Class V. *Synovial fringes.*—Five cases, with after-history of three.

Class VI. *Melon-seed bodies.*—One case.

The following questions of interest arise, and will receive a brief discussion: The nature of the conditions giving rise to internal derangement in this series; the general results of operation, and the particular results in each of the various classes; and the indications for operative interference shortly after the original injury.

It is very remarkable, though in accordance with the experience of surgeons elsewhere, that in at least thirteen cases very little deviation from the normal could be found. In some of these the cartilage was rather loose; in others it was frankly described as normal. As half the cases yielded an excellent result, we must presume that no other abnormal condition was overlooked in these at least, although in one or two cases it is possible that a loose body or synovial fringe was missed. Why knees with little or no recognisable abnormality should produce swelling and attacks of locking persisting over many months is not very evident.

In twenty cases there was an obvious tearing or loosening of one or other semilunar cartilage; in two it was the external and in eighteen the internal that was at fault. The abnormalities were very various.

In seven cases the internal cartilage was split.

In three cases the internal cartilage was not torn, but very loose.

There were one or two instances of each of the following: Anterior end torn free (2), posterior end torn free (2), external cartilage left attached only at ends (2), internal cartilage torn across middle (1), left attached only at ends (1) or only in front (1), anterior part torn away from capsule except at the extreme end in front (1).

The loose bodies were usually composed of bone and cartilage (5 cases) or cartilage only (4 cases). In one instance the body was derived from the internal semilunar cartilage ; in several cases it was noted that there was a depression on the femur from which a piece of bone and cartilage had separated. In several cases there was a loose fibrous attachment. In one knee the loose body was organising blood-clot. Once there were two bodies present, and such difficulty was experienced in getting hold of the second that it had to be left. No after-history is available. A remarkable case in which both knees were full of melon-seed bodies is not included here.

The relation to injury was very definite in nearly all cases. It is precisely stated that in nine patients the cause was a football accident, and in another the game was hockey. Probably there were others besides these.

**The Results of Operation.**—Of the whole series of forty cases, excluding one in which the clinical picture was quite unlike that characteristic of internal derangement—

| | | | | |
|---|---|---|---|---|
| In 25 | the result | is described | as | excellent. |
| „ 7 | „ | „ | „ | good. |
| „ 4 | „ | „ | „ | fair. |
| „ 4 | „ | „ | „ | bad. |

No case died as a result of the operation.

There is a remarkable and significant difference between the results in the second and third classes. Where a damaged cartilage was found and removed the operation was extraordinarily successful, and there was not a single failure. On the other hand, when a loose body was removed, there were three unsatisfactory results, and only two quite recent cases could be classed as excellent.

Six patients mention that they are now able to play football again, several are miners, and one is a sailor. One miner is able to do his work without inconvenience after removal of a cartilage from *both* knees.

GENERAL TABLE OF END-RESULTS.

| CLASS. | No. of operations. | No. of after-histories. | Excellent. | Good. | Fair. | Bad. |
|---|---|---|---|---|---|---|
| I. Cartilage normal or slightly loose .. | 13 | 8 | 4 | 2 | 1 | 1 |
| II. Cartilage torn or very loose .. .. | 20 | 17 | 16 | 1 | 0 | 0 |
| III. Loose bodies .. .. | 11 | 7 | 2 | 2 | 2 | 1 |
| IV. Notes inadequate .. | 6 | 5 | 2 | 1 | 1 | 1 |
| V. Synovial fringes .. | 5 | 3 | 1 | 1 | 0 | 1 |
| VI. Melon-seed bodies.. | 1 | 1 | 0 | 0 | 0 | 1 |
| Total .. .. | 56 | 41 | 25 | 7 | 4 | 5 |

Class I. *Cartilage normal or slightly loose.*—One of the cases in this group is a well-known professional cricketer, and was incapacitated by the frequency with which his knee "came out." He is now able to play both cricket and football without any trouble. Like several other patients, he mentions that although there is no pain or stiffness, the knee occasionally grates a little on movement.

One case suppurated, and though the result is pretty good, he gets occasional pain and stiffness. Another is able to use the knee well and gets no pain, but describes it as "tender." In another the result is less satisfactory, and for some unknown reason he gets pain when he puts his whole weight on it and has to be careful going downstairs.

One patient is quite unrelieved, and gets pain and a sensation of something in the way. He cannot work without a hinged knee-cap. It is probable that in this case something was overlooked.

Class II. *Torn or very loose cartilage.*—Here the results are very remarkable, and there can be few operations so successful. The only case out of seventeen that can be described as less than excellent says that "the result is in every way successful," but he gets a "little stiffness and a grating noise in the knee," and the leg is still weak. This is about fourteen months after the operation. Another says he gets a "stabbing pain" occasionally, but this does not hinder him from playing football and cricket.

One patient had been previously operated on elsewhere, and the internal semilunar stitched in place. Relapse took place after two and a half years, and at operation there was a longitudinal split in the middle of the cartilage. Unfortunately, he is lost sight of. His other knee had also been operated on elsewhere and the cartilage excised ; this joint was quite sound.

Class III. *Loose bodies.*—It is interesting to notice that in several of these the diagnosis was made by a skiagram before operation. In two cases, both dating from the middle of 1911, the result is excellent. Another is able to work, but gets stiffness at changes of the weather. A fourth patient gives a similar account of himself, and gets " an occasional shock of pain," but is " very pleased with it generally."

Another relapsed after six months, and the knee is now continually " coming out," although there is no pain or swelling. He is in Canada, but intends to come home for another operation.

A patient who was operated on in 1905 did quite well for two years and then relapsed. At a second opening of the joint a fresh loose body, consisting of bone and cartilage, was removed, the semilunar cartilage was torn and was also excised, and the knee showed osteo-arthritic changes.

Another gets pain, swelling and locking, with grating ; the outer side of the joint is now at fault.

It is very evident that these results are far inferior to those obtained by removing a torn cartilage. It is well known that if an intra-articular fracture takes place in a normal animal union is usually good. It is probable that loose bodies are caused by some osteo-arthritic changes, so that after operation a second fragment may be detached, or stiffness and swelling develops. If this were due merely to the friction of a mobile particle in the joints, the torn semilunar would also be followed by permanent changes.

Class IV. *Notes inadequate.*—The results in this class were satisfactory, except in two cases. In one of these it would appear that too much damage was done to the internal lateral ligament by the incision opening the joint. The result was good for seven or eight months, and then he was re-admitted because "the knee gave way." The muscles were wasted and there was lateral mobility. The ligament was tightened up and the knee fixed in plaster.

Another patient complains that her knee still aches occasionally and has "come out" twice since the operation, which was in 1905. The last trouble was in July, 1910.

Class V. *Synovial fringes.*—In one case the cartilage was removed as well as the fringe, and the result is excellent. Another patient took off his dressings, suppurated, and has a swollen, stiff knee in consequence.

A girl of twenty has been operated on twice for a knee full of synovial fringes, with swelling, but no locking. It had persisted for several months, and had once been diagnosed as tuberculosis. A year after the second operation the knee is still doing well, but the opposite joint has now become stiff and swollen.

Class VI. *Melon-seed bodies.*—Here we put a remarkable case of a man of fifty-one with osteo-arthritis of many joints of about a year's duraion. Both knees were enormously swollen and painful, and felt doughy. Movement was fairly good. The joints were opened, and an immense quantity of melon-seed bodies evacuated. The synovial membrane was thick, smooth and pale, without any fringes. The cartilages were eroded and somewhat lipped. Examination of the contents showed an excess of fibrin in the fluid; a cell-count yielded 50 per cent. polymorphonuclears, 40 per cent. endothelial cells, and 10 per cent. lymphocytes. The fluid was sterile, and did not induce tuberculosis in a guinea-pig.

The patient is still very crippled, but can get about a little with a stick.

**Early traumatic cases.**—In five cases the operation was performed within nine weeks of the injury, and this raises the question how long a stiff or swollen knee following on an accident should be treated by non-operative measures, and what are the indications for opening the joint. In one case, three weeks old, the patient could not walk, and the internal semilunar cartilage was found split. The joint swelled up after the operation and required aspiration. Possibly it had not recovered sufficiently from the original trauma. The final result is excellent.

In two cases, nine weeks and four weeks old respectively, loose bodies were discovered ; one was palpable and the other was diagnosed by the skiagram.

In two other patients thickened synovial fringes were discovered and removed eight weeks and six weeks after the accident. In one case the knee had been painful and swollen ever since ; there is no after-history. In the other there was definite locking, and the knee had to be reduced " with a jump " by the out-patient surgeon. This was the man who spoiled the result by taking off his dressings.

This is the only case which suppurated out of forty-seven knees operated on for internal derangement since the end of 1903, about which time the use of gloves became customary.

### TABLE OF CASES.

CLASS I.—*A normal or slightly loose cartilage removed.*

| Initials. | Sex. | Age. | Year. | History and Findings. | End-Results. |
|---|---|---|---|---|---|
| J. H. | M. | 32 | 1902 | Struck knee 5 months ago. Often slips out. | *Feb. 1912 :* Excellent result. No symptoms. A sailor. |
| F. F. | M. | 28 | 1903 | Knee swollen. Adhesions found and removed, as well as cartilage. | Suppurated, opened. *Nov., 1911 :* " On the whole the operation has proved successful." Occasional pain and stiffness. |
| N. B. | F. | 17 | 1903 | Knee " put out repeatedly." | (Not known.) |
| E. B. | M. | 26 | 1904 | Knee swollen since accident a year ago. | *Nov., 1911 :* Practically cured. " Very occasionally it will give a jerk." |
| A. H. | F. | 22 | 1904 | 3 years' attacks. Blood clot in synovial membrane. | (Not known.) |
| A. H. | M. | 28 | 1905 | 1 year's attacks. Inflammatory nodule also excised. | *Nov., 1911 :* " A perfect success." Plays football. |
| A. P. | M. | 22 | 1907 | 1 year's attacks, frequent displacement. Professional cricketer. | *Nov., 1911 :* " As right now as before I injured it." Cricket and football. |
| E. E. | M. | 19 | 1907 | Several years' attacks. | (Not known.) |

CLASS I. (*continued*).

| Initials. | Sex. | Age. | Year. | History and Findings. | End-Results. |
|---|---|---|---|---|---|
| M. S. | F. | 21 | 1907 | Swollen knee 6 weeks, no injury. Contains bloody fluid. Congested. | (Not known.) |
| J. K. | M. | 24 | 1908 | 3 months' history; knee swollen and often gives out. Normal cartilage removed | *Dec., 1911:* No pain or stiffness; walks and cycles. Knee tender. |
| G. E. | M. | 22 | 1910 | 9 months after wrench. Often "gives way." Normal cartilage removed. | *Dec., 1911:* Pain, weakness, sense of "something in the way." Cannot work without hinged knee-cap. |
| W. H. | M. | 31 | 1910 | Two football accidents. Swelling and attacks of locking for many months. | *Dec., 1911:* Some pain when he puts whole weight on it. Care in going downstairs. |
| W. H. | M. | 22 | 1910 | 3 months' history; cannot walk since. | (No after history.) |

CLASS II.—*Cartilage, torn, displaced, or very loose. Removed.*

| Initials. | Sex. | Age. | Year. | History and Findings. | End-Results. |
|---|---|---|---|---|---|
| A. M. | M. | 29 | 1901 | Old injury recently renewed. Pain and stiffness. Cartilage displaced. | *Dec., 1911:* "My knee is in good condition." No pain or swelling. Can run as well as ever. |
| T. W. | M. | 41 | 1902 | 7 years' clicking, locking and falling down. Gouty. Tag partly detached. | (No after-history.) |
| D. W. | M. | 22 | 1904 | Recurrent swelling 9 months. Cartilage torn across middle. Alar ligament of mucosa also removed. | *Nov., 1911:* "As well as ever it was." Can run, jump and kick |
| H. W. | M. | 27 | 1905 | Many attacks, 4 years' history. Front of cartilage broken loose. | *Dec., 1911:* "Is all right." Works as miner. Second knee operated on in 1910. |
| J. B. | M. | 22 | 1906 | Front part of cartilage lying loose across joint. | *Nov., 1911:* "My knee is quite strong." Footballer. No pain or stiffness. May ache when weather changes. |
| J. L. | M. | 32 | 1906 | Often laid up for 3 years. Posterior half of cartilage rolled up in front of condyle. | *Nov., 1911:* "A great success in every way." A little "sticking pain" if he walks far. |

Class II. (*continued*).

| In-tials. | Sex. | Age. | Year. | History and Findings. | End-Results. |
|---|---|---|---|---|---|
| E. E. | M. | 39 | 1906 | History 18 months. Cartilage loose and thickened. | *Nov., 1911 :* "I am doing very well . . . quite satisfied." No pain; swells after hard work. |
| W. B. | M. | 52 | 1906 | Locking attacks 12 years. Flexion limited. Cartilage ¾-inch mobile in front. | *Nov., 1911 :* "Made me a new man and a young man." No symptoms. Heavy work. |
| H. T. | M. | 22 | 1908 | Numerous attacks. External cartilage broken loose except at ends. | *Nov., 1911 :* "I can move it as freely as ever." Cricket and football. No symptoms. |
| W. B. | M. | 25 | 1908 | Frequent attacks for 3 years. Cartilage only attached back and front. | *Nov., 1911 :* "My knee is all right." No pain or swelling. |
| E. L. | M. | 35 | 1909 | Knee stiff and painful since twist 5 months ago. Cartilage split; tag partly detached. | *Nov., 1911 :* "As fit as ever I was . . . Not inconvenienced the least bit." Miner. |
| R. W. | M. | 40 | 1910 | History 6 months. Loose tag partly detached from cartilage. Felt before operation. | *Dec., 1911 :* "In every way successful." Leg still weak and some grating, however. |
| J. H. | M. | 39 | 1910 | Weak and swollen since twist 2 months ago. Posterior end of cartilage displaced forwards. | *Dec., 1911 :* "Knee is all right." No symptoms. |
| H. W. | M. | 46 | 1910 | Unable to walk since blow 3 weeks ago. Snap felt by patient. Layer of cartilage found peeled up in front. | Synovitis followed; aspirated. *Dec., 1911 :* "Is all right," can run. Miner. Both knees operated on. |
| A. H. | M. | 35 | 1910 | Repeated attacks of locking and swelling, 4 months. Longitudinal split in cartilage. | (No after-history.) |
| G. J. | M. | 32 | 1910 | Locking and swelling 4 years. Front of cartilage detached except at tip. | *Dec., 1911 :* "In every way satisfactory." Plays games. A stabbing pain occasionally. |
| W. M. | M. | 46 | 1910 | Twisting and clicking 10 years. Cartilage lying free across joint, only attached in front | *Dec., 1911 :* No pain, swelling or stiffness. |
| G. P. | M. | 23 | 1910 | Sensation of loose body with pain and clicking 7 months. External cartilage torn loose in middle. | *Dec., 1911 :* "As fit as ever." |

CLASS II. (*continued*).

| Initials. | Sex. | Age. | Year. | History and Findings. | End-Results. |
|---|---|---|---|---|---|
| D. K. | M. | 36 | 1911 | 3 years. Cartilage stitched in place elsewhere. Recurred $2\frac{1}{2}$ years later. Cartilage found split. | (No after-history.) |
| H. W. | M. | 27 | 1911 Mar. | 6 weeks, pain since twist. Cartilage split longitudinally. | *Dec., 1911:* "As well as the other." |

CLASS III.—*Loose bodies. Removed.*

| Initials. | Sex. | Age. | Year. | History and Findings. | End-Results. |
|---|---|---|---|---|---|
| A. P. | M. | 16 | 1901 | Loose body felt projecting 4 weeks since injury. Bone and cartilage from femur. | *Jan., 1912:* Relapsed 6 months after; often locking. Coming from Canada for operation. |
| W. H. | M. | 39 | 1902 | History 9 years. Nodule of cartilage ($\frac{1}{2} \times \frac{1}{4}$ inch), from femur, fibrous attachment. A second could not be extracted; was left. | (No after-history.) |
| J. P. | M. | 21 | 1904 | Attacks of locking; loose body lelt; 10 weeks. Cartilage and bone, size of halfpenny. | *Nov., 1911:* "Able to work as usual"; stiffness at change of weather. |
| E. P. | M. | 45 | 1905 | Loose body many years. Removed. | Well for 2 years; then pain and swelling. Opened. Torn cartilage, removed; also large loose body; osteo-arthritis of joint. (No further history.) |
| J. P. | M. | 49 | 1905 | Pain and inability to extend since fall 9 months ago; swelling near patella. Bony lump with fibrous attachment. | *Nov., 1911:* Occasional pain, swelling, locking and grating. Pain now on outer side. |
| M. R. | F. | 15 | 1905 | Lump felt since a blow. Organised blood-clot, size of halfpenny. | (No after-history.) |
| H. T. | M. | 41 | 1907 | Locking; loose body obvious; 4 months since a twist. Cartilage and bone. | (No after-history.) |
| F. O. | M. | 20 | 1908 | Locking and cracking, 18 months. Part of semilunar cartilage separated as a loose body. | (No after-history.) |

CLASS III. (*continued*).

| Initials. | Sex. | Age. | Year. | History and Findings. | End-Results. |
|---|---|---|---|---|---|
| D. B. | M. | 32 | 1911 (May) | Locking, 6 months. Cartilaginous. Internal semilunar normal, but removed. | *Dec., 1911 :* " Am very pleased with it generally," but much aching and stiffness at weather changes. |
| A. H. | M. | 22 | 1911 (June) | Lump felt; locking; 8 months. Cartilaginous, with fibrous attachment. | *Jan., 1912 :* Excellent result. |
| T. H. | M. | 23 | 1911 (July) | Twisted knee 9 weeks ago; swollen and stiff. Skiagram shows loose body. Bone and cartilage ($1 \times \frac{3}{4}$ inch). | *Dec. 29, 1911 :* " No pain or swelling "; can run; no symptoms. |

CLASS IV.—*Notes inadequate; operation performed.*

| Initials. | Sex. | Age. | Year. | History and Findings. | End-Results. |
|---|---|---|---|---|---|
| J. H. | M. | 24 | 1904 | Swollen three times; football accident. | *Nov., 1911 :* " As strong as the other." Footballer. |
| G. E. | M. | 19 | 1905 | Loose cartilage, removed. | Returned a year later with wasting of muscles, lateral mobility and giving ways of knee. Internal lateral ligament tightened by operation. |
| E. T. | F. | 17 | 1905 | History 2 years. Cartilage removed. | *Nov., 1911 :* " Still aches occasionally." " Slipped out " twice since. Can run and kneel. |
| A. B. | M. | 33 | 1907 | Frequent locking, 1 year. Cartilage removed. | *Nov., 1911 :* " Got allright." Occasional pain and stiffness, but satisfactory. Died insane. |
| J. G. | M. | 27 | 1907 | Eight years' history. Cartilage removed. | (No after-history.) |
| W. E. | M. | 35 | 1907 | Repeated locking for 18 months. Cartilage removed. | *Nov., 1911 :* " Has been a great success." No trouble except a slight soreness after heavy work. |

CLASS V.—*Synovial fringes removed.*

| Initials. | Sex. | Age. | Year. | History and Findings. | End-Results. |
|---|---|---|---|---|---|
| A. L. | F. | 40 | 1902 | Pain and swelling since fall 8 weeks ago. Loose thick fringe of alar ligament removed. | (No after-history.) |
| E. F. | M. | 27 | 1903 | Six years' pain and locking. Tag of synovial membrane catching in joint removed. | Suppurated furiously. Many subsequent operations to drain. |
| G. W. | M. | 63 | 1910 | Visible lump; locking, 6 years. Normal cartilage and hypertrophied alar ligament removed. | *Dec., 1911:* "Perfect cure." |
| S. M. | M. | 19 | 1911 (March) | Knee "out" repeatedly for 6 weeks; reduced by surgeon. Fibro-fatty synovial nodule removed. | Patient removed dressings; suppurated. *Dec., 1911:* Painful, stiff, swollen. |
| M. W. | F. | 20 | 1911 (Jan.) | Knee swollen 2 months; no locking; looked like tubercle. Full of villous synovitis; cleared out at two operations; second operation transpatellar, in March. | *Dec., 1911:* Quite well, but 3 weeks ago the other knee began to swell. |

CLASS VI.—*Osteoarthritis.*

E. S., male, aged 51, was operated on in 1910. He suffered from osteoarthritis of many joints; the fingers were spindled. For about a year the knees had been very swollen and rather painful; movement was fairly free. They felt soft and doughy, and there was soft grating. At operation immense masses of soft white melon-seed bodies were evacuated. Synovia thick, smooth, white; no fringes. Cartilage eroded and somewhat lipped.

The fluid contained fibrin in excess. It was sterile, and inoculation of a guinea pig for tubercle was negative. A cell-count showed endothelials 40 per cent., polymorphonuclears 50 per cent., lymphocytes 10 per cent.

In January, 1912, there is no great improvement, there is still pain and stiffness, but he can get about a little with a stick.

